# Supplementary material for: Transcriptome Analysis Reveals Vimentin-Induced Disruption of Cell–Cell Associations Augments Breast Cancer Cell Migration
Source: Cells. 2022 Dec 13;11(24):4035. doi: 10.3390/cells11244035 (PMC9776984; doi:10.3390/cells11244035)
Supplement: Supplementary file 1 [file cells-11-04035-s001.zip › DEGs and enrichment analysis.zip!/DEGs and enrichment analysis/WT_VIMvsVector KEGG/ALL/WT_VIMvsVector_ALL.pathway_map.zip!/WT_VIMvsVector_ALL.pathway_map/src/hsa00230.html]

hsa00230


- hsa:107

- Up regulated genes

ENSG00000164742(1.87205666251591)

- hsa:956

- Down regulated genes

ENSG00000168032(-2.54891241689973)

- hsa:956

- Down regulated genes

ENSG00000168032(-2.54891241689973)

- hsa:956

- Down regulated genes

ENSG00000168032(-2.54891241689973)

- hsa:956

- Down regulated genes

ENSG00000168032(-2.54891241689973)

- hsa:956

- Down regulated genes

ENSG00000168032(-2.54891241689973)

- hsa:956

- Down regulated genes

ENSG00000168032(-2.54891241689973)

- hsa:107

- Up regulated genes

ENSG00000164742(1.87205666251591)

Close
